# Advancing Ceramic Membrane Technology for Sustainable Treatment of Mining Discharge: Challenges and Future Directions

**DOI:** 10.3390/membranes15040112

**Published:** 2025-04-03

**Authors:** Seyedeh Laleh Dashtban Kenari, Saviz Mortazavi, Sanaz Mosadeghsedghi, Charbel Atallah, Konstantin Volchek

**Affiliations:** 1 CanmetMINING, Natural Resources Canada, 555 Booth Street, Ottawa, ON K1A 0G1, Canada; sanaz.mosadeghsedghi@nrcan-rncan.gc.ca (S.M.); charbel.atallah@nrcan-rncan.gc.ca (C.A.); konstantin.volchek@nrcan-rncan.gc.ca (K.V.); 2 Office of Energy Research and Development, Natural Resources Canada, 580 Booth Street, Ottawa, ON K1A 0E4, Canada; saviz.mortazavi@nrcan-rncan.gc.ca

**Keywords:** ceramic membrane, treatment, mining discharge, acid mine drainage, nanofiltration, microfiltration, ultrafiltration, vacuum membrane distillation

## Abstract

Mining discharge, namely acid mine drainage (AMD), is a significant environmental issue due to mining activities and site-specific factors. These pose challenges in choosing and executing suitable treatment procedures that are both sustainable and effective. Ceramic membranes, with their durability, long lifespan, and ease of maintenance, are increasingly used in industrial wastewater treatment due to their superior features. This review provides an overview of current remediation techniques for mining effluents, focusing on the use of ceramic membrane technology. It examines pressure-driven ceramic membrane systems like microfiltration, ultrafiltration, and nanofiltration, as well as the potential of vacuum membrane distillation for mine drainage treatment. Research on ceramic membranes in the mining sector is limited due to challenges such as complex effluent composition, low membrane packing density, and poor ion separation efficiency. To assess their effectiveness, this review also considers studies conducted on simulated water. Future research should focus on enhancing capital costs, developing more effective membrane configurations, modifying membrane outer layers, evaluating the long-term stability of the membrane performance, and exploring water recycling during mineral processing.

## 1. Introduction

‘Mining and Quarrying’ is one of the oldest industries in Canada and provides raw materials for most of the production sectors. This sector experienced a significant growth rate of 48% from 2000 to 2018 [1], making it one of the most rapidly expanding industrial sectors in Canada.

Water is used for a variety of purposes in mining industries, including milling, floatation, leaching, hydrometallurgical separation techniques, such as staged precipitation, solvent extraction, ionic exchange, membrane separation, electrowinning, cooling of equipment, slurry transport, dust suppression and human consumptions at mine sites [2,3].

In coal mining industries, the effluent stream, for instance, contains considerable amounts of dissolved salts [4]. Stone cutting mines produce a slurry of very small particles/colloids with poor settling properties [5], which can cause an increased turbidity in the discharge water, leading to serious damage to its ecosystem. In addition, mining industries are predominantly located in remote areas, where groundwater is their main water source, having high salinity levels of up to 200,000 ppm. Moreover, mining operations may also use seawater, such as in the Michilla Copper Mine in Chile, and wastewater, as reported in the Cadia Gold Mine in Australia, either alone or in combination with mine dewatering water, recycled effluent, or mine water runoff [2]. Therefore, mining effluents, with superior turbidity, salinity, and acidity, need to be treated before disposal to the environment. In addition, the treated water can be recycled and reused, providing a sustainable approach for water management in mining industries.

Acid mine drainage (AMD) is a profoundly adverse effect of mining activities, causing significant environmental issues due to its low pH levels (2–4), presence of hazardous metals, and high sulfate concentrations (0.1–20 g/L). The corrosive nature of AMD would provoke the solubility of toxic metals, resulting in generation of AMD with elevated level of dissolved constituents, such as Al, As, Ca, Cd, Co, Cu, Fe, Mg, Mn, Ni, Pb, Se and Zn [2,6]. The AMD generation is primarily related to the oxidation of sulfide containing mineral ores, particularly pyrite ore (FeS_2_), because of its oxidation when exposed to oxygen, water, or microorganisms [7]. Fe(II) is commonly the most abundant element in AMD, which readily reacts with dissolved oxygen, producing yellowish water with a high level of suspended particulates. Therefore, to protect human health and the environment, many government agencies are forcing the mining industry to treat all effluents in accordance with guidelines and standards before their discharge to the environment. By 1993, Canada possessed a cumulative amount of 1.8 billion metric tons of sulfide tailings that had the capacity to induce AMD, a figure that is likely to be significantly greater presently [8]. Typically, mines in Canada have a lifespan of 15 to 20 years, although the management of waste and treatment of AMD are expected to be ongoing indefinitely [9]. As a result, there are more than 10,000 actives, closed, and abandoned mines in Canada, necessitating remediation.

Several technologies have been applied to treat mining discharge, depending on the treatment objectives. Active and passive treatments are the two major categories of AMD remediation. The active treatment methods include chemical precipitation, oxidation, coagulation, flocculation, sand filtration, adsorption, ion exchange, and membrane technology. The passive treatment of AMD uses constructed wetlands for biological treatment, limestone drains for chemical treatment, and sulfate-reducing bioreactors [10]. This approach is better suited to abandoned mines than the continuous AMD flow. It has the advantage of low maintenance and operating costs, as well as not requiring continuous chemical injection [2]. Furthermore, it has few environmental impacts, and the produced waste is denser and more stable than the sludge generated during chemical treatment [11]. Despite these benefits, passive systems suffer from several drawbacks that hinder their widespread industrial application. For instance, these systems have limited capacity for producing alkalinity and increasing the pH of AMD [2]. Therefore, metals such as Zn and Mn cannot be effectively removed [12]. Passive systems are generally less effective than active methods while requiring a much longer processing time.

Several factors, such as water composition, pH, and treatment cost, dictate the choice of mine discharge treatment. Mining effluents traditionally undergo the chemical precipitation process, which involves the addition of chemical reagents followed by a solid–liquid separation step [13]. Ferrous sulfate, ferric chloride, alum, and lime are coagulants commonly used in chemical conditioning steps. Among these coagulants, iron coagulation appeared to be the most effective, especially for arsenic removal [14]. Amaral et al. [15] described the application of this technology and the separation via dissolved air floatation. Another common technique for this purpose is coagulation, followed by filtration. These two processes cannot be effectively applied separately and have been considered a two-in-one process [16]. The removal of heavy metals via precipitation of their sulfide form has received considerable interest in the treatment of mining discharge [17,18]. The formation of toxic H_2_S under low pH conditions is, however, a drawback of this process. Although the precipitation process results in the removal of most undesirable ions, including toxic metals, it may yield an effluent with high salinity [19]. In addition, it produces a large amount of sludge with a high water content, for which dewatering and disposal pose immense problems. Moreover, the produced sludge has no economic value, owing to the difficulty of recovering the metals. Therefore, the waste should be disposed of in landfills with a special design to avoid the re-dissolution and migration of toxic metals, which can dominate the operational costs and make the process unsustainable [2]. Alternatively, technologies that can remove dissolved salts, such as evaporation, ion exchange, adsorption, and membrane filtration, have gained remarkable interest.

Despite the low cost of natural evaporation, it is a prolonged process with a significant footprint. In contrast, energy-driven evaporation is vastly more effective but substantially more expensive [19]. The ion exchange technique for metal removal has gained popularity in mining industries over the past few decades [19]. Commonly employed ion exchange media include ion exchange membranes, and anionic and cationic resins. Buzzi et al. [20] evaluated the application of anion- and cation-exchange membranes for AMD treatment. Electrodialysis was found to be very effective for recovering water from AMD, with a removal capacity of more than 97% for contaminants; however, the precipitation of iron at the surface of the membrane and the blockage of the membrane’s pores substantially reduced the efficiency of the process. As a result, the drawbacks associated with this process, such as iron fouling, organic substance adsorption, and bacterial contamination, significantly increase the process’s operational costs, remarkably reducing the efficiency of the ion exchange membranes [21].

Previous studies have assessed the removal of various metals from cation exchange resins and sulfate ions from anion exchange resins [22,23]. This integrated process was revealed to generate purified water from contaminated mine discharge and AMD. The mining sector has commercialized this technology. The GYP-CIX is a highly effective technique that adopts fluidized bed ion-exchange to remove calcium and sulfate from gypsum-saturated water [24].

Environmental and Remedial Technology Holdings (Earth) Pty Ltd. developed a patented and commercially available ion exchange technique to recover uranium and sulfate ions from AMD [25]. The main drawback of the ion exchange process is the considerable quantity of brine that is produced during the regeneration step. The high quantity of gypsum and toxic metals in the brine may render it unsuitable for reuse. Moreover, the disposal of brine poses significant challenges.

Adsorption is a highly effective method for wastewater treatment. This method can remove metals from mine water even at very low concentrations [26,27]. Typically, this technique is highly effective in handling low metal concentrations, but its efficacy is restricted when faced with high metal levels. Regardless, the selection of the sorbent medium greatly influences the effectiveness of this process.

As mentioned above, the consequences of untreated mine water on human health, the environment, and aquatic life are very disastrous. In this regard, the choice of an appropriate treatment option with no or limited environmental impacts became crucial. This necessitates the development of advanced treatment technologies to diminish the above-mentioned limitations. Over the past few decades, membrane technology has found increasing application in water and wastewater treatment, distillation, and desalination. The application of microfiltration (MF), ultrafiltration (UF), nanofiltration (NF), and reverse osmosis (RO) membranes in wastewater treatment has been growing exponentially to meet the increasingly stringent regulations for effluent discharge and water reuse. While several studies have begun to address mine water treatment using polymeric membranes, very few have focused on ceramic membranes. The industrial application of ceramic membranes for mine discharge treatment remains restricted due to a lack of scientific research and detailed information. This is while the application of ceramic membranes for the treatment of mining discharge has gained considerable attention owing to their superior chemical (e.g., oxidants such as ozone and chlorine) and thermal resistance and mechanical strength over traditional polymeric membranes [28]. The superior durability of ceramic membranes leads to better abrasion resistance over polymeric membranes, which is a key advantage in treating mine-impact wastewaters that often contain abrasive and coarse particles. In addition, ceramic membranes possess lower operational costs while offering operation under higher flux compared to polymeric ones [29]. The capacity for back flushing, as well as the ease of cleaning and disinfection, makes them a compelling option [30,31].

This review focuses primarily on the application of ceramic membranes for mining discharge treatment and investigates the potential adoption of membrane distillation for comparable objectives. Additionally, an effort was made to identify the gaps in the existing body of literature to guide future research and practical implementation.

## 2. Overview of Ceramic Membrane Material and Configuration

There are two main types of ceramic membranes that are commercially available for the purpose of wastewater treatment, the oxide ceramic membranes that are made of Al, Si, Ti or Zr oxides and silicon carbide (SiC), which cover the range from MF to NF. Different materials exhibit varying chemical and hydrothermal stability, as well as surface charges in the solution that must be chosen appropriately for a specific application [32]. The chemical stability of oxide ceramic membranes decreases in the order of TiO_2_, ZrO_2_, Al_2_O_3_, SiO_2_, and their hydrothermal stability diminishes in the order of ZrO_2_, Al_2_O_3_, TiO_2_, and SiO_2_ [33]. In recent years, SiC ceramic membranes have additionally gained attention in water treatment, and they have been shown to also possess significant chemical and thermal stability [34]. The high capital cost of ceramic membranes compared to polymeric ones is another key challenge for many industries. The expensive raw materials, such as metal oxides, and the need for high sintering temperatures render ceramic membranes uneconomical unless they are operated at high flux values. Consequently, numerous researchers have focused on the development of ceramic membranes using alternative low-cost raw materials to reduce the cost of the membranes. Some examples are natural minerals and industrial wastes, such as clay, silty marls, kaolin, zeolite, bauxite, quartz sands, bentonite, cordierite, apatite, perovskite, coal fly ash, coal gangue, blast furnace slag, rice husk ash, pozzolan, etc., [35,36]. The ceramic membrane composed of these natural minerals and industrial wastes was estimated to be significantly cheaper than conventional metal oxide membranes. In addition, in recent years, sustainable fabrication techniques have been developed to reduce the environmental impact and cost of ceramic membranes. For example, food waste-derived pore-forming agents have been successfully adopted to fabricate ceramic membranes for wastewater treatment applications, demonstrating the potential of low-cost materials in membrane manufacturing [37].

**Figure 1:**
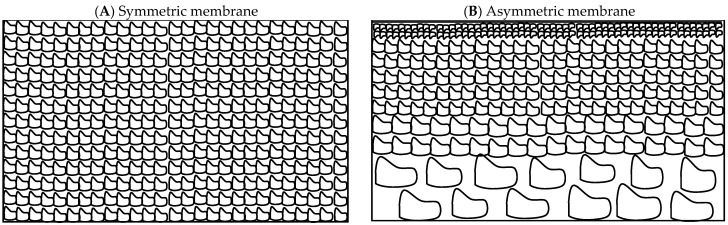
Schematic of cross-section of a (**A**) symmetric and (**B**) asymmetric membrane.

Another category of ceramic membranes includes symmetric membranes made of a single material with the same pore size or asymmetric membranes made of a porous support layer and a thin skin top layer with the desired pore size (Figure 1). Different layers of asymmetric membranes for industrial applications can be made of the same material, integral membranes, or of different materials, composite membranes [29]. These layers possess successively smaller pore sizes as materials with finer particle sizes are used to fabricate them, and the top-most or selective layer can further be chemically modified to promote better fouling resistance.

Ceramic membranes must provide a large surface area for large-scale applications, which can be configured into packages known as membrane modules. There are three general configurations of ceramic membranes for industrial applications: (1) flat-sheet, (2) tubular, and (3) hollow-fiber. Tubular and hollow-fiber membranes are more favorable for applications in the mining industry because of their higher packing density and mechanical stability, easier sealing of the elements, and better capability to handle high crossflow velocities compared to flat-sheet membranes [32]. The fragility of the membrane, however, limits the optimization of the packing density of the ceramic modules to reduce the process footprint [32].

**Figure 2:**
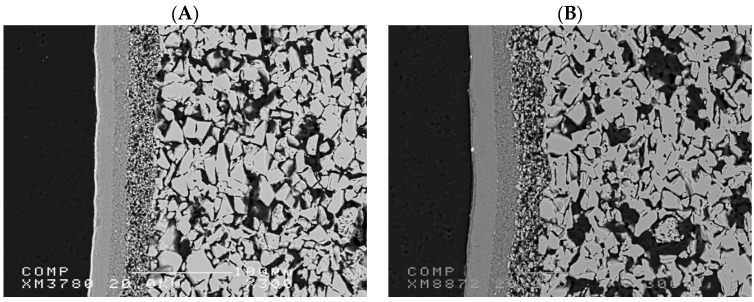
SEM image of the cross-section of commercially available asymmetric ultrafiltration ceramic membranes from Inopor: (**A**) TiO_2_ membrane and (**B**) Al_2_O_3_ membrane.

Figure 2 illustrates the SEM image of a TiO_2_ and an Al_2_O_3_ commercially available asymmetric ultrafiltration ceramic membranes from Inopor. The membranes have similar structures with an active layer of 5 nm and a porosity range between 30 and 55%. The microstructure of the ceramic membranes reveals a highly porous bulk structure with an interconnected network of grains. The presence of a denser top layer suggests an asymmetric structure, crucial for selective filtration performance. The observed porosity of the support layer ensures high permeability, while the compact layer at the surface provides effective separation.

## 3. Overview of Membrane Fouling

Despite the importance of membrane separation for water treatment, its application in the treatment of mine-impacted waters has been limited due to membrane fouling and abrasion, which not only affects the productivity of the membrane by decreasing its permeability but may also deteriorate the quality of the water generated. Membrane fouling is an inevitable phenomenon caused by the adsorption of organic and inorganic substances and microorganisms into the membrane pores and/or their accumulation on the surface of the membrane. This can lead to the blockage of the membrane pores and/or the formation of the cake layer, respectively, resulting in additional resistance to flow. As such, four membrane fouling mechanisms have been identified: complete blocking, standard blocking, intermediate blocking, and cake filtration. Various approaches have been undertaken to mitigate ceramic membrane fouling, including pretreatment, membrane surface modification, membrane cleaning, i.e., back flushing and chemical cleaning under high temperature and acidic/basic conditions, magnetization, ultrasonics, and nanobubbles [29,38].

The hydrodynamics of the membrane system and the properties of the feed constituents, the membrane, and the solution phase can affect membrane fouling [38]. Membrane foulants can be classified into four groups: inorganic, organic, colloidal, and biofoulants. Inorganic fouling or scaling is caused by the accumulation of inorganic precipitates (e.g., CaSO_4_ and CaCO_3_) within the membrane pores and/or on the surface of the membrane due to their solubility limit. Colloidal fouling is caused by different particles and colloids, such as algae, certain organic matter, and clay, which can be readily removed from the membrane surface by backwashing and air scrubbing unless the size of the colloids is small relative to the membrane pore size. Organic fouling is generated by organic solutes of various molecular weights ranging from a few thousand to 1 million Dalton, such as humic substances and proteins. Biofouling is caused by the formation of biofilm on the membrane surface [39]. In the treatment of mine-impacted waters, inorganic fouling by coarse mineral-based particles is most likely to occur, requiring the use of membranes with abrasion resistant surface properties. This is in contrast to the treatment of other types of wastewater, such as municipal water systems, where less abrasive organic particles are mainly responsible for membrane fouling. Various membrane materials may have different fouling potentials owing to their distinct surface properties. Previous studies revealed that polymeric membranes may have a higher potential for organic fouling compared to ceramic membranes due to their lower hydrophilicity [40]. In addition, ceramic membranes of various materials may also present different fouling potentials based on their pore size as well as surface roughness and charge [40]. Although severe fouling may occur during filtration using ceramic membranes, previous research has demonstrated that chemical cleaning might completely restore the membrane’s original permeability [41].

Mining discharges have a wide range of chemical complexity and diversity, including various concentrations of toxic or heavy metals in particulate or dissolved form, which can notably affect the fouling buildup. Although the commercially available ceramic membranes are 3–10 times more expensive than the polymeric ones, the application of ceramic membranes remarkably reduces the operating cost, especially in aggressive operational environments such as AMD treatment [42]. The improved operating costs are attributable to higher operating fluxes achievable by ceramic membranes, and a lower propensity to membrane fouling and degradation compared to polymeric membranes. These advantages can potentially help offset the initially higher capital cost of ceramic membranes. A better understanding of fouling mechanisms by analyzing the interactions between the membrane and the foulants present in mining effluents can result in developing an appropriate pretreatment strategy under optimal conditions, which can further alleviate operational costs.

In addition to the previously mentioned factors, fouling behavior is significantly affected by the intrinsic properties of ceramic membranes, such as pore size, tortuosity, and the presence of structural defects [43]. Internal pore blockage, as one of the most severe fouling phenomena, can be mitigated using membranes with narrow and uniform pore size distributions, while increased tortuosity may enhance resistance to foulant intrusion by promoting more complex flow paths [44]. Moreover, the design of membrane modules, i.e., cross flow vs. dead end, also impacts the extent of membrane fouling as well as its behavior [45]. For example, crossflow configurations in tubular or flat-sheet modules generate higher shear forces along the membrane surface, reducing the accumulation of particulate and organic matter, i.e., cake formation. These structural considerations are critical to optimize membrane longevity and effectiveness, especially in high-strength waste streams such as mining effluents and AMD.

It is very important that future research address the fouling potential of mine discharge water for the economic analysis of the ceramic membranes for this application. The following sections summarize the application of ceramic membrane for MF, UF, and NF mining discharges, as well as the potential application of vacuum membrane distillation (VMD) for this purpose.

## 4. Application of Ceramic MF/UF Membrane for Treatment of Mining Effluent

Low-pressure membranes, such as MF and UF, have been increasingly employed in wastewater treatment facilities as an alternative to conventional filtration processes. The nominal pore size of the MF and UF membranes range from 0.1 to 10 μm and 1 to 100 nm [46], respectively. Impurities in water have been classified in the following categories: (1) settleable solids (>100 μm), (2) supra-colloidal solids (1–100 μm), (3) colloidal solids (0.001–1 μm), and (4) dissolved solids (<0.001 μm) [47]. Therefore, an appropriate choice of MF/UF membrane can effectively remove suspended particles, colloids, macromolecules, and oil from water [48,49]. These membranes can be applied as a standalone process [50] or in hybrid integration with other processes [51,52], such as pre-treatment for NF and RO membranes [53]. Due to the low transmembrane pressure of the MF/UF systems, their operational costs are much lower than those of the NF and RO membranes; however, their application is limited to the removal of particulate and colloidal constituents, and macromolecules from water. Accordingly, the dissolved metal ions in mining discharge and AMD must be precipitated by oxidation and/or sulfide/hydroxide addition prior to the MF/UF membrane. In this section, the important parameters influencing the MF/UF membrane fouling by inorganic particles or colloids, as the main suspended solids in mine effluent and AMD, are briefly discussed. It was then attempted to review the scientific literature and commercial reports on mine discharge treatment.

Sieving or straining is the primary mechanism responsible for the separation of particulate and colloidal substances from a liquid using MF/UF membranes. In this regard, constituents larger than the membrane pore size are retained at the surface, while smaller ones pass through the membrane. Removal efficiency might be further affected by adsorption during the early stages of filtration [46]. In addition, the formation of a cake layer of large compounds could reduce the passage of small particles through the membrane’s pores during the later stages of filtration [46]. Accordingly, the removal efficiency of each contaminant may vary over the course of filtration.

The deposition of particulates and colloids within the membrane pores and/or on the surface of the membrane also leads to the membrane fouling. Therefore, pore plugging and cake filtration are the two dominant fouling mechanisms in MF/UF processes. In addition, the surface charge of the membrane and the suspensions in water and the electrostatic interactions between them could also impact the membrane performance with respect to particulate removal as well as membrane fouling. Accordingly, the repulsive interactions between the colloids and the membranes may prevent them from entering the pores, resulting in the removal of colloids smaller than the pore size of the membrane [54]. Moreover, when the colloids are highly charged, the strong repulsion between colloids increases the permeability of the deposited cake layer (i.e., reduction in membrane fouling) due to the larger apparent diameter of the colloids [55,56]. On the other hand, the size of inorganic particles or colloids in water may be significantly affected by their surface charge, which is in turn controlled by the background water chemistry (pH, ionic strength, and salt valency) [51,57]. Therefore, the size of inorganic particles or colloids may increase several orders of magnitude under conditions in which the background chemistry of water encourages the destabilization of colloids (e.g., high ionic strength). These aggregated particles (flocs) are filtered to form a cake layer on the membrane’s surface with high porosity and minimal resistance. This is not only due to the large size of the flocs but may also be related to their fractal structure. Previous research demonstrated that the fractal structure of the flocs affects the permeability of the deposited cake layer, thereby influencing the extent of membrane fouling [58,59].

Dashtban Kenari et al. [51] compared the performance of ceramic and polymeric UF membranes for iron and manganese removal from groundwater. Nonetheless, the dissolved iron and manganese were oxidized by potassium permanganate prior to MF/UF membranes. Both membranes removed >99% of iron and manganese from water. The results also illustrated that the size, surface charge, and fractal dimensions of oxidized iron and manganese in water, which were controlled by the pH, ionic strength, and salt valency of the background water could substantially affect the extent of membrane fouling. In addition, the extent of fouling caused by these inorganic particles and colloids under different conditions was quantitatively identical for both ceramic and polymeric membranes; however, the compressed cake formed during operation with ceramic UF membrane was almost completely removed by backwashing with permeate and air scouring, while for the case of polymeric membrane, 78–98% of fouling under different conditions was physically irreversible. Noteworthy is the low mechanical stability of the polymeric membrane, which does not allow backflushing with air. Instead, backwashing with permeate followed by a forward flush with air was executed for the polymeric membrane, unable to appreciably restore membrane permeability.

**Figure 3:**
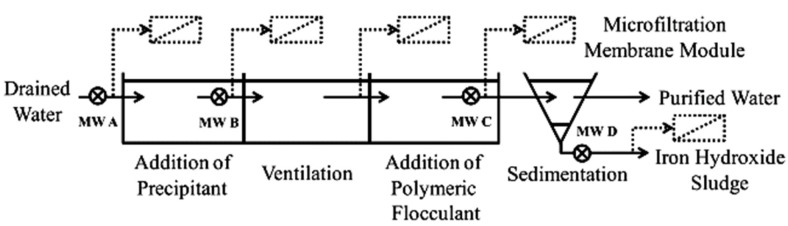
Schematic presentation of a water purification system and possible integration of a MF membrane module. Reproduced with permission from [60].

There is a lack of published research on mining effluent treatment using different ceramic MF/UF systems. As shown in Figure 3, in a pilot-scale application, Meschke et al. [60] investigated the use of a rotating disk ceramic MF membrane as an alternative to a conventional filtration process that involves a chemical conditioning step and sedimentation for AMD treatment from an opencast lignite mine. Rotating membrane disks create high shear rates on the membrane surface to minimize fouling and thus the flux decline.

AMD commonly contains elevated levels of dissolved iron, mainly ferrous iron, due to its strongly acidic condition [53]. The effectiveness of a ceramic MF membrane (α-Al_2_O_3_; 2.0 um pore size) was investigated with respect to iron removal and membrane fouling. The results showed that applying the ceramic membrane in crossflow mode as a downstream process, after chemical conditioning with 5% lime milk and 0.1 *w*/*w* polymeric flocculant, can guarantee a high steady-state flux of 590 LMH with a rejection efficiency of >99.9%. The addition of polymeric flocculant increased the permeability of the membrane by a factor of 5.9. Promising results were also obtained via dead-end microfiltration mode of iron hydroxide sediments for sludge dewatering applications. Despite the much higher solid content of sediment compared to pretreated water (1.5% vs. 0.4%), a relatively high steady-state flux of 220 LMH and > 99.9% rejection efficiency were achieved. As expected, direct filtration of AMD by ceramic MF membrane was not able to appreciably reduce the concentration of iron in the effluent due to the dominance of dissolved iron in AMD, which was not removed by the MF membrane despite developing a layer of iron hydroxide on the membrane surface. After filtration, the membrane was chemically cleaned for 1 h using a 0.01 mol/L HNO_3_ solution. The permeability of the cleaned membrane to those of brand-new ones decreased by a factor of 2.3–2.5, indicating insufficient chemical cleaning procedures.

Laitinen et al. [5] studied the removal of fine suspended solids from an open stone cutting mine wastewater using a silica-modified alumina (pore size of 100 nm) and a ɣ-alumina (pore size of 10 nm) ceramic UF membrane. Both membranes removed more than 99% of total suspended solids (TSS), and the concentration of iron (<0.03 mg/L) and manganese (<0.07 mg/L) in the effluent met the discharge limit. Using a 100 nm membrane pore size, the permeate flux continuously declined even after 24 h of filtration, indicating the continuous fouling of the membrane. In the case of the 10 nm ɣ-alumina membrane, stable flux was achieved due to its smaller pore size, which prevented the small particles from penetrating inside the membrane pores. Nevertheless, the pure water permeability of this membrane was low (~55 LMH/bar) due to the tightness of the membrane; however, the permeability was only reduced by 7–12% during the operation without backflushing. Backflushing was unable to recover membrane permeability in this case. Chemical cleaning, by soaking the membranes in a 0.1 mol/L HNO_3_ solution overnight, only recovered 3–4% of the membranes’ permeability, implying that either the applied cleaning procedure was insufficient or most of the fouling was chemically irreversible. It might also be related to the irreversible compaction of the UF membrane structure due to the high transmembrane pressure applied [53]. Further research shall be carried out to investigate the potential application of a simple pretreatment step, such as inline flocculation, to alleviate membrane fouling while maintaining a high permeate throughput. Table 1 summarizes the studies evaluating the utilization of MF/UF ceramic membranes for mine discharge treatment.

**Figure 4:**
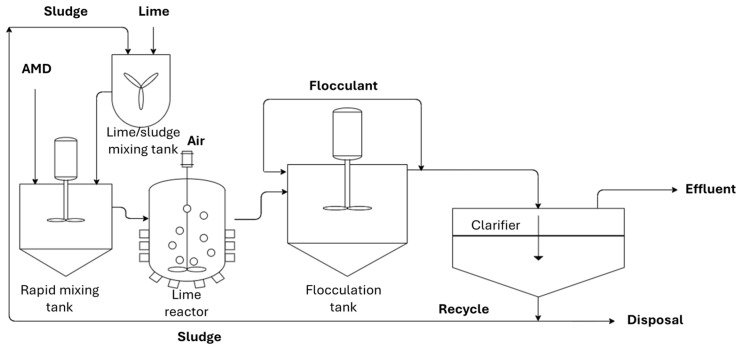
High-density sludge clarifier.

As a field-scale case study, Stewart compared three types of AMD active treatment systems at Blackhawk, Colorado, consisting of a high-density sludge (HDS) clarifier, a polymeric MF membrane, and a ceramic MF membrane [42]. The HDS clarifier system (Figure 4) was applied to recycle the sludge formed by lime precipitation, decreasing the overall process footprint and the utilization of lime.

**Figure 5:**
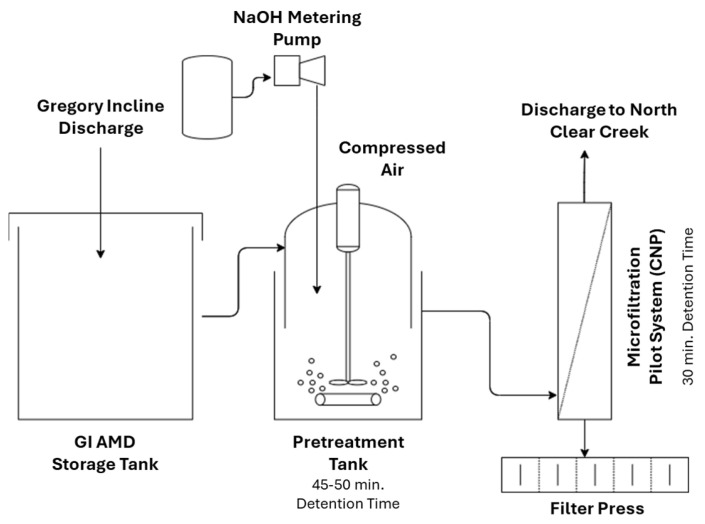
Schematic diagram of the MF membrane treatment system.

Figure 5 illustrates a schematic diagram of the MF membrane treatment system. The polymeric and ceramic membranes were both microporous with similar pore sizes and were operated under the same transmembrane pressures. The first treatment step was the conversion of dissolved heavy metals into precipitates that can be effectively removed by the MF membrane with a 0.2 μm pore size. In this regard, the pH of the wastewater was adjusted between 8.5 and 9.5 via a hydroxide precipitation step. The wastewater was then pumped through the crossflow MF membrane system. It was found that the treated water through the MF membrane has a lower pH than the water treated by the HDS clarifier, thus meeting the discharge requirements on a consistent basis. In addition, smaller footprints, lower chemical consumption, labor costs, and power costs were other notable advantages of replacing the clarifier system with an MF membrane. The main difference between the polymeric and ceramic membrane systems was the lifespan of the membranes. The tubular polymeric membranes originally used at the Blackhawk had a lifespan of 6–9 months, while the ceramic membranes installed in 1995 were still in service until 2013, indicating the durability of the ceramic membrane [42].

Another commercial-scale case study is the ceramic MF system installed in 2009 at the Upper Blackfoot Mining Complex in Montana, USA [61]. Compared to the HDS clarifier system installed at this facility, the ceramic MF membrane could operate in very acidic conditions, which is an advantage for AMD treatment.

In a practical treatment study, Bakalár et al. [62] studied an integrated microfiltration-reverse osmosis (MF-RO) process for the treatment of contaminated mining water. The microfiltration system was a tubular ceramic membrane with an active α-Al_2_O_3_ layer resistant up to 150 °C, allowing a pH range of 0.5 to 13.5 [62]. The water produced after the reverse osmosis stage was demineralized water, which was remineralized using Semidol, thereby meeting the discharge quality limit [62]. Ceramic MF membranes have not only been applied for the treatment of mining discharge but have also been adopted for the recovery of minerals from these streams. In this respect, Kumar et al. [63] investigated the application of a ceramic MF membrane for the recovery of copper sulfate nanoparticles (CuSNPs) from AMD. To recover highly pure CuSNPs, an integrated bioprecipitation and MF process were applied. Prior to dead-end microfiltration, pretreatment of the bioprecipitate was conducted using probe sonication (for 90 min) and resulted in a very high separation efficiency of CuSNPs (92%) [63]. Compared to the other pretreatment methods tested, probe sonication was the most effective method for impurity removal, likely due to its use of high-power ultrasounds, followed by cell lysis using a French press, centrifugation and heating, and bath sonication [63]. The separation of the nanoparticles by microfiltration was achieved at 172 kPa and ambient room temperature (25 ± 2 °C) [63]. The characterization of the CuSNPs showed excellent crystallinity, size, shape, and purity, with polycrystalline products of a size range of 5–10 nm [63].

The review of previous commercial applications of MF/UF ceramic membranes in treating severe mining effluents demonstrates the advantages of this technology. The high capital cost of ceramic membranes and the lack of detailed information on process efficiency, however, hindered their widespread application. Based on the review above, it is evident that previous research has not conducted in-depth studies to determine the impact of mine water chemistry, inorganic particle and colloid characterization, and pretreatment strategies on the performance of ceramic MF and UF membranes. Moreover, future studies should focus on analyzing the fouling mechanism to mitigate its effects and explore the influence of different membrane materials, such as polymeric versus ceramic MF/UF membranes, on the reversibility of fouling during the filtration of mine effluents and AMD. Furthermore, more research should address the design of a hybrid process combining ceramic MF/UF membranes and other physico-chemical treatment technologies to enhance the process’s sustainability and reduce the need for sludge management, as demonstrated in conventional processes.

## 5. Application of Ceramic NF Membrane for Treatment of Mining Effluent

NF membranes offer a viable alternative technology for treating mining effluent and AMD. Typically, ceramic NF membranes range in molecular weight cut-off (MWCO) from approximately 500 to 1000 Da, making them suitable for the removal of organic molecules, natural organic matter, and multivalent ions [4]. Numerous studies have explored the use of polymeric NF membrane in the mining sector [64,65,66,67,68,69], but only a few studies have investigated the application of ceramic membrane [4,70,71], despite its exceptional durability. Due to the limited research on the application of ceramic NF membranes in the mining sector and the critical need to understand the mechanisms of ion removal via ceramic NF membranes, this section first reviews the literature on the removal of various ions from synthetic water. It concludes with an analysis of the available literature on the application of NF in the mining sector.

Sulfate removal is the major concern in AMD treatment that can be present at concentrations as high as 20 g/L [4]. Van Gestel et al. [72], Weber et al. [73], and Wadekar and Vidic [4] tested the sulfate rejection of ceramic NF membranes with an active layer of TiO_2_ and MWCO of ~500 Da. Synthetic solutions of monovalent and divalent species including Na_2_SO_4_, as a sulfate source, were prepared to feed the membranes in these experiments. Sulfate retention with the ceramic NF membrane increased to >90% at pH values above 10 [72,73], which is comparable to the common sulfate retention by a polymeric NF membrane in the entire pH range [4,72,73]. Nevertheless, AMD is characterized by high acidity, with pH ranges between 2 and 4 [64], at which less than 70% sulfate removal was reported [4,72,73]. The rejection of dissolved ions with the ceramic NF membranes is mainly controlled by the membrane surface charge, which is, in turn, dictated by the solution pH, salt type and salt concentration [4,72,73]. The lowest salt retention was observed at isoelectric point (IEP) of the membrane, where an ion transport is only impeded by the ion size. It is worthwhile mentioning that although the IEP of TiO_2_ membrane lies between pH 6 and 7 [64,72,73,74], the apparent point of zero charge may significantly change with background salt(s) [72,73,74]. For instance, the TiO_2_ membrane is always positively or negatively charged, under the entire pH range, in 10 mM CaCl_2_ or Na_2_SO_4_ solution, respectively [72]. Therefore, removal of ions from AMDs at a specific pH value could significantly vary depending on their composition. In general, retention of different ions with ceramic NF membrane is significantly associated with the zeta potential of the membrane in the solution and the repulsion of co-ions [72]. Accordingly, high sulfate removal could be achieved at alkaline pH condition while high calcium removal could be obtained at an acidic pH [72].

Van Gestel et al. [72] compared the membrane rejection efficiency for 0.14 and 1.42 g/L sulfate in the feed solution. Their results indicated that sulfate retention/rejection declined as the feed water sulfate concentration increased. For instance, at pH 7.0, increasing the sulfate concentration from 0.14 to 1.42 g/L reduced its rejection by 22% (67% vs. 45%). This was attributed to the reduction in the thickness of the electrostatic double layer surrounding the pores of the membrane’s top layer under a higher ionic strength condition, which resulted in lower sulfate retention [72]. In addition, salt retention is determined by the ratio of the membrane charge to feed concentration, which declines with an increment in feed concentration [73]. In accordance, it was demonstrated that CaCl_2_ retention significantly decreased with increasing its concentration in the feed solution, from 0.5 to 3.0 g/L [75]. In addition, the permeability of the NF membrane is also reduced by increasing salt concentrations due to the increased osmotic pressure. In contrast, Wadekar and Vidic [4] observed an increase in sulfate rejection from synthetic feed water, from 17 to 68%, as the sulfate concentration increased from 0.5 to 10 g/L without specifying the pH of the synthetic water. This was explained by the adsorption of sulfate on the surface of the membrane, resulting in further resistance against ion transport. This discrepancy in the literature might be attributed to the different concentration ranges used in these studies (0.14 to 1.42 g/L vs. 0.5 to 10 g/L). Further research is required to clarify the impact of sulfate concentration on the rejection efficiency of the ceramic NF membrane.

In addition to the abovementioned parameters, transmembrane pressure (TMP) could also affect the salt retention of a ceramic NF membrane. Wang et al. [75] indicated that the retention of AlCl_3_ and CaCl_2_ slightly increased with raising the TMP from 2.0 to 5.0 bar. This is in accordance with the results reported by Van Gestel et al. [72] and Weber et al. [73] for NaCl and Na_2_SO_4_ retention. This can be explained by the dominant ion transport mechanism of the NF membrane under different pressures. Ion transport is mainly governed by diffusion under low pressure, while convection and electromigration play important roles under high-pressure conditions.

Wang et al. [75] developed a γ-Al_2_O_3_/α-Al_2_O_3_ hollow fiber ceramic membrane with MWCOs ranging from 6000 to 1400 Da for salt retention applications. In general, multivalent ions, such as Fe^3+^ and Al^3+^, were better retained by the membrane than monovalent ions, such as Na^+^ (>90% vs. <30% with the 4000 Da membrane). For ions with the same valence, retention is determined by the hydrated radii or diffusion coefficient of the ion. Accordingly, the ion with a lower diffusion coefficient has a larger hydrated radius and, thus, a higher retention [76]. The lowest retention was observed for the sulfate ion (<10%) with the smallest hydrated radius.

Arsenic is one of the most challenging contaminants in mining effluent and AMD. It occurs in water as inorganic salts and organic forms with different molecular sizes and charges. The pH of water controls the electric charge of certain arsenic species, thereby affecting the performance of NF membranes for arsenic removal. Accordingly, it is necessary to discriminate the concentration of different arsenic compounds because they have different rejection characteristics in NF. Urase et al. [77] revealed that at pH 3, 5, and 7, most of the arsenite (As(III)) is in neutral solute form because of its high pKa value (9.1), while at pH 10, arsenite is dominantly present in monovalent anion form. Arsenate (As(V)) is either present in monovalent or divalent anion at pH 3 to 10. Accordingly, it can be expected that arsenate is better removed by the NF membrane than arsenite. In accordance, previous researchers demonstrated a higher rejection efficiency of arsenate than arsenite with a polymeric NF membrane [77,78]. In order to enhance arsenite removal, Sen et al. [79] suggested a pre-oxidation step prior to the polymeric NF membrane to convert arsenite to arsenate. It was indicated that pre-oxidation by permanganate increased arsenic removal from 50 to 63% to 97 to 100% [79]. In spite of several studies that have been conducted on arsenic rejection by polymeric NF membranes, there has been a lack of information on the fate of arsenite and arsenate in the ceramic NF membrane process.

Wadekar and Vidic [4] compared the performance of ceramic and polymeric NF membranes for treating abandoned coal mine drainage in southwestern Pennsylvania. The asymmetric ceramic membrane that was used had an active layer of amorphous TiO_2_ with a MWCO of about 500 Da. The polymeric membrane comprises polypiperazine amide with a MWCO of ~200–400 Da. AMD was pretreated with aeration and MF for iron removal (60 mg/L), thereby preventing severe membrane fouling. Several dissolved elements, including aluminum, arsenic, barium, manganese, nickel, selenium, and strontium, as listed in Table 1, were present in the pretreated AMD and required removal prior to discharge. In the case of ceramic membranes, retention of all ions increased with an increasing permeate recovery up to 75%, resulting in the removal of 55 to 67% of divalent cations. Changing the permeate recovery, however, did not have any significant impact on the rejection of multivalent cations with the polymeric membrane, which was always more than 96%, except for aluminum (80–90%). Additionally, the polymeric membrane rejected 93% of sulfate, whereas the ceramic membrane achieved sulfate removal of up to 62.8%. A very low arsenic rejection of 20% and 33% was reported using ceramic and polymeric membranes, possibly due to the presence of uncharged species (arsenite). Reducing the feed pH from 7.8 to 4.0 led to an increase in ionic rejection and permeability for the ceramic membrane, as it affected the charge of the active layer. The same results were reported by Van Gestel et al. [72], indicating an increased rejection of monovalent and divalent ions under lower pH conditions using a TiO_2_ NF membrane. These results delineated that at pH 4.0, charge (Donnan) exclusion contributed to ionic rejection rather than size exclusion. The largest increase in rejection was observed for arsenic (81.5% increase) and selenium (45% increase). In contrast, rejection of all ions except arsenic was reduced or remained constant using a polymeric membrane. In this case, arsenic removal increased by only 5%.

**Table 1:** Summary of the investigations evaluating the application of MF/UF/NF ceramic membranes for AMD and mine discharge treatment.

Feed Source and Characteristics	Ceramic Membrane Characteristics	MWCO/Pore Size	Manufacturer	Operation Condition	Permeability (at Steady State)	Pre-Treatment	Rejection Efficiency	Ref.
AMD (opencast lignite mining) Fe_total_: 220 mg/L Solid content: 0.3%	Material: α-Al_2_O_3_ compact rotating disk ID: 25 mm OD: 152 mm Thickness: 4.5 mm Clean water permeability: 1392 LMH/bar	2.0 μm	Novoflow GmbH	Crossflow Feed flow: 3.38 L/min TMP: 1.2 bar pH: 2.5 T: 25 °C	141.7 LMH/bar	Coating of membrane surface by a layer of iron hydroxide	70.4%	[59]
AMD (opencast lignite mining) Fe_total_: 290 mg/L Solid content: 0.4%				Crossflow Feed flow: 3.42 L/min TMP: 1.2 bar pH: 6.0 T: 25 °C	83.3 LMH/bar	5% lime milk	Fe: >99.9%	
AMD (opencast lignite mining) Fe_total_: 330 mg/L Solid content: 0.4%				Crossflow Feed flow: 4.13 L/min TMP: 1.2 bar pH: 7.7 T: 25 °C	491.7 LMH/bar	5% lime milk + 0.1 *w*/*w* Koaret PA 3230	Fe: >99.9%	
AMD (opencast lignite mining) Fe_total_: 5000 mg/L Solid content: 1.5%				Dead-end Feed flow: 0.30 L/min TMP: 1.9 bar pH: 7.8 T: 25 °C	115.8 LMH/bar	5% lime milk + 0.1 *w*/*w* Koaret PA 3230 + static thickening	Fe: >99.9%	
Stone cutting mine wastewater TSS: 485 mg/L Turbidity: 365 NTU COD: 27 mg/L P_total_: 0.3 mg/L Fe_total_: 17.5 mg/L Mn_total_: 0.7 mg/L	Material: Silica-modified Al_2_O_3_ Plate type	0.1 μm	N/A	Crossflow CFV: 4.5 m/s TMP: 1.1 bar pH: 6.8 T: 20 °C	Steady state not reached	None	TSS: >99% Turbidity: >99.9% COD: <18 mg/L (DL) P_total_: >99% Fe_total_: >99.9% Mn_total_: >90%	[5]
					127.3 LMH/bar	Biological treatment		
	Material: ɣ-Al_2_O_3_ Plate type	0.01 μm			51.8 LMH/bar	None	TSS: >99% Turbidity: >99.8% COD: <18 mg/L (DL) P_total_: >95% Fe_total_: >99.8% Mn_total_: >91%	
AMD	Tubular ceramic membrane	0.2 μm	N/A	Crossflow Feed flow: 38–1325 L/min CFV: 3 m/s TMP: 0.35 bar Operation pressure: 2.41 bar pH: 8.5–9.5	N/A	NaOH addition + aeration	Turbidity: 0–2 NTU As: 66.4% Cd: >99.9% Ca: 78.3% Cr: >99.3% Cu: >99.9% Pb: >99.9% Mn: 99.8% Ni: 99.8% Ag: 99.8% Zn: >99.9%	[60]
AMD ${SO}_{4}^{-2}$: 645.9 mg/L Cl: 97.8 mg/L Na: 108.9 mg/L Ca: 151.8 mg/L Mg: 29.7 mg/L K: 4.3 mg/L Mn: 1.2 mg/L Fe: <0.02 mg/L Sr: 1.7 mg/L Ba: 76.7 μg/L Al: 50.5 μg/L Ni: 38.5 μg/L As: 70.0 μg/L Se: 55.2 μg/L	Material: fused Al_2_O_3_ with active surface layer of TiO_2_ Single channel tubular membrane ID: 6 mm Length: 500 mm	500 Da (~1 nm)	Cerahelix	Crossflow Feed flow: 5.68 L/min CFV: 3.35 m/s TMP: 35 bar pH: 7.8 T: 25 °C	0.8 LMH/bar	20–24 h aeration + 0.22 um MF membrane	${SO}_{4}^{-2}$: 63% Cl: 7% Na: 36% Ca: 60% Mg: 68% K: 38% Mn: 65% Sr: 60% Ba: 56% Al: 42% Ni: 67% As: 20% Se: 46%	[4]
				Crossflow Feed flow: 5.68 L/min CFV: 3.35 m/s TMP: 35 bar pH: 4.0 T: 25 °C	1.6 LMH/bar		${SO}_{4}^{-2}$: 68% Cl: 11% Na: 40% Ca: 63% Mg: 70% K: 45% Mn: 65% Sr: 62% Ba: 59% Al: 43% Ni: 67% As: 36% Se: 63%	
				Crossflow Feed flow: 5.68 L/min CFV: 3.35 m/s TMP: 35 bar pH: 7.8 T: 25 °C Antiscalant: 15 mg/L	0.4 LMH/bar		${SO}_{4}^{-2}$: 87% Cl: 26% Na: 66% Ca: 80% Mg: 85% K: 71% Mn: 80% Sr: 80% Ba: 78% Al: 45% Ni: 83% As: 60% Se: 70%	
Mine discharge Ca^2+^: 134.27 mg/L Mg^2+^: 130.38 mg/L Cl^−^:123.08 mg/L SO_4_^2−^: 134.69 mg/L Nitrate: 108.06 mg/L As: 5 mg/L Cu: 5 mg/L Fe: 5 mg/L Ni: 5 mg/L Available particle sizes: 0: 0.5–1.2 mm I: 0.5–2.5 mm II: 2.0–4.5 mm III: 4.0–7.0 mm	Ceramic membrane (MF) MF Material: active layer of α-Al_2_O_3_ on a rigid porous base Resistant up to:150 °C pH range: 0.5–13.5 Shape: tubular form (inner diameter 7 mm, length 25 cm, and effective membrane area of 50 cm^2^)	0.1–0.5 μm	Semidol Porosity: 14.4% Bulk density: 1.1–1.2 t.m^−3^	Original pH: 7.18 conductivity: 693 mS.m^−1^ T: 10.5 °C After MF pH:6.40 conductivity: 756 mS.m^−1^ T: 20.5 °C After RO pH: 7.59 conductivity: 0 mS.m^−1^ T: 19.7 °C Remineralized pH: 8.72 conductivity: 0 mS.m^−1^ T: 19.7 °C	N/A	N/A	After treatment Ca^2+^: 32.06 mg/L Mg^2+^: 10.94 mg/L Cl^−^: 8.43 mg/L SO_4_ ^2−^: 3.45 mg/L Nitrate: 0.84 mg/L As: 0 mg/L Cu: 0 mg/L Fe: 0 mg/L Ni: 0 mg/L	[62]
Synthetic AMD CuCl_2_·2H_2_O: 50 mg/L Na_2_SO_4_: 1470 mg/L Supplemental mineral salt medium NH_4_Cl: 1.0 g/L KH_2_PO_4_: 0.5 g/L Na_2_SO_4_: 1.47 g/L CaCl_2_·2H_2_O: 0.1 g/L FeSO_4_·6H_2_O: 0.289 g/L Na_3_C_6_H_5_O_7_: 0.3 g/L EDTA: 0.3 g/L Yeast extract: 1.0 g/L	Membrane material: Kaolin: 14.45 wt% Quartz: 26.59 wt% Ball clay: 17.58 wt% Pyrophyllite: 14.73 wt% Feldspar: 5.6 wt% Effective membrane diameter: 42 mm Effective membrane thickness: 4 mm	1.01 μm	Low-cost ceramic membrane prepared based on the composition reported by Monash and Pugazhenthi (2011)	Dead-end Microfiltration to obtain pure CuSNPs from the bio precipitate at a constant applied pressure (172 kPa)	N/A	Probe sonication: highest separation efficiency of CuSNPs (92%) Other pretreatment methods tested (more to less effective separation of CuSNPs): cell lysis using a French press, centrifugation and heating, bath sonication	N/A	[63]
Recycle water from Canadian oil sands Cations Li^+^: 0.1–0.2 mg/L Na^+^: 220.0–360.0 mg/L K^+^: 11.8–18.6 mg/L Mg^2+^: 12.3–16.0 mg/L Ca^2+^: 25.1–34.1 mg/L Ba^2+^: 0.1–0.2 mg/L Anions F^−^: 1.3–3.3 mg/L HCO^−3^: 349.0–509.0 mg/L Cl^−^: 103.0–167.0 mg/L Br^−^: 0.2–0.4 mg/L SO_2_^−4^: 163.0–268.0 mg/L NO^−3^: 0.1–2.7 mg/L Components TSS: 13.0–305.0 mg/L TOC: 31.0–134.0 mg/L Hardness (as CaCO_3_): 72.0–151.0 mg/L Silica (SiO_2_): 2.7–20.5 mg/L Total Silicon, Si: 2.7–9.6 mg/L Total Boron, B: 1.3–2.4 mg/L Total Sulfur, S: 61.0–109.0 mg/L	Commercial titania Ceramic NF Membrane Unit Membrane surface area: 1.3 m^2^/element Pure water flux range (at 1 bar): 15–20 LMH Tmax 400 °C Pressure stability: ≥60 bar	Mean pore size: 0.9 nm MWCO: 450 Da	Inopor	Crossflow TMP (highest): 13.3 bar pH: 7.7–8.4 T: 6–36 °C Test condition: constant flow or constant TMP mode Operation: 50% stage cut for approximately 75 days (around 1800 h) Average recycle process water flow: 7.0 m^3^/h	1–10 LMH/bar	N/A	Cations K^+^: 63% Na^+^: 62% Li^+^: 60% Ba^+^: 73% Ca^2+^: 68% Mg^2+^: 65% Anions Cl^−^:42% NO^3−^: Br^−^: 67% F^−^: 54% HCO^3−^:61% SO4^2−^: 69% Components TOC: 92% TSS: 100% Hardness (as CaCO_3_): 66% Silica (SiO_2_): 58% Total Silicon, Si: 58% Total Boron, B: 58% Total Sulfur, S: 70%	[70]
Synthetic AMW solutions (mimicking those from the Iberian Pyrite Belt in Huelva province (Southwest of Spain)) 2 scenarios: one with Fe(III) one without pH: 1.0, 1.5 Al(III): 600, 1800 (mg/L) Fe(III): 500, 125 (mg/L) Ca(II): 25 mg/L Cu(II): 40 mg/L Zn(II): 46 mg/L REEs(III): 60 mg/L	TiO_2_ tubular ceramic membrane Area: 44.92 cm^2^ Internal diameter: 6.5 mm Thickness: 2 mm Active layer of TiO_2_ supported on Al_2_O_3._ Flat-sheet polymeric MPF–34 (proprietary layer) Area: 140 cm^2^	TiO_2_ ceramic membrane: 1nm Polymeric MPF–34: 200 Da	Ceramic membrane: Fraunhofer IKTS Polymeric membrane: Koch Membrane Systems (MPF–34)	Cross flow Ceramic: Cross flow velocity (cfv): 3.5 m/s TMP: 6–13 bar pH: 1–12 T: 25 °C Polymeric MPF–34: Cfv: 0.7 m/s TMP: 6–20 bar pH: 1–12 T: 25 °C	Ceramic: 9–13 LMH/bar Polymeric MPF–34: 0.6–3.6 LMH/bar	Pre–filter cartridge: 100 μm, polypropylene	TiO_2_ metal rejections: <60%, (highest rejections for trivalent transition metals) MPF–34 metal rejections: 80% independent on the concentration of the major components (Al(III) and Fe(III))	[71]

In this study, the greatest fouling occurred at 75% permeate recovery for both polymeric and ceramic membranes and was dominated by gypsum (CaSO_4_) scaling; however, less fouling was observed for the ceramic membrane than that for the polymeric one. The permeability of the ceramic and polymeric NF membranes decreased by 13.6% and 16.2%, respectively. Chemical cleaning with NaOH and citric acid/hydrochloric acid improved membrane permeability but reduced ionic rejection, implying a slight increase in the effective membrane pore size of both membranes. The addition of antiscalant significantly enhanced the rejection of all ions by the ceramic membrane while having only a marginal impact on the ionic rejection of the polymeric membrane, except for arsenic. It was reported that arsenic rejection improved by 200% and 141% with the addition of antiscalant for the ceramic and polymeric membranes, respectively. However, the permeability of both membranes decreased by 40% owing to the formation of a more complex and gel-like scale [4]. The type and dosage of the antiscalant were not optimized in this study. For the operation of full-scale plants, intensive pilot-scale tests should be conducted to determine the best antiscalant with the optimum dosage.

In a long-term pilot study, Motta Cabrera et al. [70] tested a commercial titania unit to reduce the concentration of undesired cations and anions, TSS and total organic carbon (TOC), as listed in Table 1, in recycled water from a Canadian oil sand mine. The system was operated at a 50% stage cut (proportion of the feed flow that is being separated or processed by the system in each stage of the operation) for approximately 75 days and was fed with an average water flow of 7.0 m^3^/h. A strong correlation was observed between the specific flux and rejection, with the highest mass rejections occurring at the lowest specific flux values [70]. Preference was observed in the rejection of both cations and anions with high charge densities, with the divalent cations experiencing the largest rejections (70%) [70]. It is worth noting that the titania membrane was negatively charged under the pH conditions tested. In this regard, it can be inferred that the cations were rejected at high charge densities due to adsorption to the membrane, while the anions were rejected owing to the electrically repulsive interactions with the same-charged membrane. In addition, the TSS and TOC were rejected at around 100 and 92%, respectively, likely due to the sieving mechanism [70].

López et al. [71] compared two acid-resistant NF membranes, ceramic TiO_2_ and polymeric MPF-34, for the treatment of synthetic AMD. The synthetic AMD was formulated to mimic the composition of the sulfur-rich effluents from the Iberian Pyrite Belt in Huelva Province, Southwest Spain. The experiments were conducted with a polymeric flat-sheet membrane in a crossflow test cell (GE SEPA™ CF II) with a spacer-filled feed channel and a tubular ceramic membrane placed on a stainless-steel module (Fraunhofer IKTS) [71]. The experimental conditions are listed in Table 1. The results demonstrated that the polymeric MPF-34 membrane rejected around 80% of metal (independent from the concentration of the main components, Al(III) and Fe(III)), whereas the TiO_2_ membrane provided < 60% rejection, with the highest rejection occurring for trivalent transition metals [71]. The observed rejection performance of the ceramic membrane could be explained by dielectric effects; thus, the chemical properties of the TiO_2_ layer played a significant role. Additionally, ceramic NF membranes with smaller pore sizes need to be developed to reduce the contribution of convective flow to ion transport. This might be achieved by altering the surface properties of the ceramic membrane, thereby reducing the effective pore size and increasing the surface charge density.

Novel amine-functionalized ceramic-supported composite membranes, P-60S (2000 Da) and P-60S-EDTA (polyethyleneimine (PEI) and EDTA-modified PEI functional layers; 1000 Da), were constructed by Roy et al. [80] and tested for the removal of As (V), Cr (VI), and Cu (II) [80]. The removal of the metal ions by both membranes is strongly influenced by the pH of the solution, despite the higher rejection rate of the P-60S-EDTA membrane (99.82% removal for Cu (II), 96.75% for As (V), and 97.22% for Cr (VI)) [80]. These results are likely associated with the lower effective pore size of the P-60S-EDTA membrane, as well as its higher complexation capacity and permeability [80]. Additionally, the presence of the EDTA-Cu(II) complex created resistance towards the passage of other heavy metal ions, improving the rejections of As (V) and Cr (VI) [81]. Building on this work, Roy et al. [81] developed ceramic-supported polymeric (CSP) composite membranes to explore the removal of cationic and anionic heavy metals from aqueous solutions, namely Ni (II), Cd (II), Pb (II), Zn (II), As (V), and Cr (VI). GPCu0 (pristine), GPCu0.5 (surface modified with 0.5 wt% copper chloride solution), and GPCu1 (surface modified with 1 wt% copper chloride solution) membranes were prepared for the rejection of these metals [81]. High rejection of cations was observed at low feed concentrations (1 mg/L) for GPCu0 (92.6–99.8%), which were further improved using GPCu0.5 (95.8–98.4%) and GPCu1 (96.8–99.9%) membranes [81]. At higher feed concentrations, multiple removal mechanisms (including chelation with surface functional groups, charge repulsion, and steric hindrance) increased the overall rejection efficiency of the GPCu0.5 membrane, with the highest removal rates occurring at 10 mg/L [81]. With the anionic heavy metal studies, the GPCu0.5 membrane showed excellent rejections of both As (V) and Cr (VI) even at elevated pressures, likely due to interactions with the amine surface functional groups of the membrane. In contrast, the surface of the GPCu1 membrane (higher saturation of Cu^2+^ ions) caused steric hindrance towards the passage of the larger Cr (VI) ions [81]. Additionally, the rejection of the small As (V) ions was shown to decrease with increasing pressure. The GPCu0.5 membrane achieved an excellent removal (>95%) of all heavy metals under all tested conditions [81].

Based on the aforementioned results, it is important to note that charge exclusion is the dominant separation mechanism, in addition to the size exclusion and dielectric exclusion phenomena that govern the high efficiency of a NF membrane. Accordingly, active layer surface modification of the ceramic membrane could help to enhance the rejection of ionic species. Additionally, from the review of past studies, it is evident that the quality of water and the type and concentration of background salt could significantly affect the rejection efficiency of the NF membrane. Despite this fact, there are only a few studies concerning the removal of ions from AMD and mining discharge using ceramic NF membranes. In addition, to the best of our knowledge, despite the importance of NF membrane modification, there is a lack of previous studies investigating the effect of surface modification on the removal of metals from mine-impacted water. Future research efforts should focus on investigating the application of various ceramic NF membranes in mining effluent treatment, as well as determining the long-term sustainability of this process. The goal of these efforts should be to demonstrate at pilot-scale that ceramic membranes are capable of maintaining high operating fluxes when treating real field samples of mine water. In such long-term studies, efforts should be made to improve the longevity of ceramic NF membranes in treating mine-impacted wastewater. This would involve the application of ceramic MF/UF membranes as pre-treatment to remove suspended solids, allowing the NF membranes to solely focus on the targeted removal of dissolved species like heavy metals and sulfates. Furthermore, there is a need for research on the development of novel surface-modified ceramic membranes that make use of specialized hydrophilic coatings to mitigate interactions between ceramic membrane selective layers and various foulants found in mining effluents, as a means of fouling remediation.

## 6. Application of Ceramic Membrane in VMD for Treatment of Mining Effluent

Membrane distillation (MD), an emerging thermally driven membrane separation process, can be applied to treat high-salinity water, such as seawater, groundwater and mine discharge, with greater efficiency compared to RO owing to its near-complete salt rejection even at high concentrations [82]. In addition, lower fouling propensity was also reported for MD systems compared to RO [82]. Furthermore, RO systems could not satisfactorily reject boron, a compound commonly present in mine waste, in its boric acid form [83]. Nevertheless, MD has thus far not attained widespread use as a stand-alone technology in water treatment. It has, however, been extensively studied in the context of integration with established industrialized technologies like RO and multi-effect distillation (MED) to develop hybrid processes. The reason for this process intensification is that MD is limited to applications where heat is readily available, preferably in the form of waste heat to improve its economic sustainability [84].

MD is a membrane-based technology that requires heat to drive separation. A membrane that allows water vapor to pass through while being impermeable to liquid water is the essential element in the MD process. Accordingly, the appropriate membrane should be hydrophobic with high liquid entry pressure (LEP) and have high porosity (80%), narrow pore size distribution (0.1–0.6 μm), low thickness (70–178 μm), low thermal conductivity (0.15–0.45 W/m K), low surface energy (20–30 × 10^3^ N/m) and high surface tension [85,86]. The hydrophobic nature of the membrane not only prevents feed solution from entering the pores, but also assists the generation of a liquid-vapor boundary layer. The mass transfer driving force is the water vapor pressure gradient across the membrane that is mainly related to the difference between the temperatures of the feed solution and the permeate. Thus, theoretically, the MD process completely rejects all non-volatile substances [87]. Small organic compounds and dissolved gases in the feed water would pass through the membrane and contaminate the permeate stream. In addition, the presence of alcohols and surfactants in the feed water could reduce the feed surface tension resulting in membrane wetting. Therefore, pre-treatment or post-treatment might be required to meet the desired water quality [86,88].

Polymeric membranes, such as polytetrafluoroethylene (PTFE), polypropylene (PP) and polyvinylidene fluoride (PVDF), are commonly applied for MD, due to their inherent hydrophobicity, which influences the LEP as well as the largest feasible membrane pore size [89]. However, it is difficult to control the pore structure and geometry of polymeric membranes, which are also critical parameters for developing effective MD membranes. In addition, the real-world application of this technology for the treatment of highly concentrated reject and other wastes streams may necessitate membrane exposure to harsh thermal and chemical environments. Polymeric membranes are also susceptible to issues related to membrane compression and deterioration, which can severely affect flux and separation performance. Accordingly, ceramic membranes were first tested in MD applications two decades ago [90] and have been gaining popularity due to their excellent thermal and chemical stabilities; however, modification of the hydrophilic nature of the ceramic membranes to hydrophobic is required. Two approaches have been applied to modify ceramic membranes, thus making them hydrophobic for MD application. The first approach is to create a rough surface with a highly hydrophobic structure by generating pillars and accumulation of air pockets on the membrane surface [91,92]. The second approach is chemical modification by a grafting agent, which possesses a low surface free energy [93,94]. Hendren et al. [95] evaluated a select number of promising surface treatment agents, such as perfluorodecyltriethoxysilane, trimethylchlorosilane, or trichloromethylsilane, to render hydrophilic ceramic membranes hydrophobic. Although both perfluorodecyltriethoxysilane and trichloromethylsilane induced sufficiently high hydrophobicity on alumina membranes, perfluorodecyltriethoxysilane treated membranes had a higher steady-state water flux, which made it a more suitable agent for MD application.

MD has been applied in various configurations with respect to the permeate side arrangement, namely direct contact membrane distillation (DCMD) [95,96], air gap membrane distillation (AGMD) [94,97], sweeping gas membrane distillation (SGMD) [98,99] and vacuum membrane distillation (VMD) [93,100].

**Figure 6:**
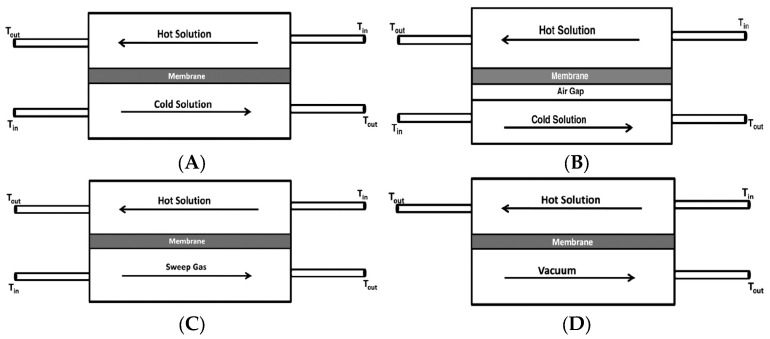
Schematic of various MD configurations: (**A**) DCMD, (**B**) AGMD, (**C**) SGMD, and (**D**) VMD.

In all configurations, as schematically shown in Figure 6, hot feed solution is in direct contact with the hot membrane feed side surface, where evaporation takes place. In DCMD (Figure 6A), the vapor condenses inside the membrane pores. It is the simplest and the most commonly applied configuration. The main inconvenience of this system is the heat loss by conduction [86]. In AGMD, as depicted in Figure 6B, stagnant air is placed between the membrane and the condensation surface, thus, reducing heat loss by conduction. This design, however, creates additional resistance to mass transfer, which is the drawback of this design. The schematic of SGMD is shown in Figure 6C, in which the vapor at the permeate side of the membrane is swept by an inert gas to condense outside the membrane module. Therefore, the heat loss is reduced, and mass transfer coefficient is enhanced. This design necessitates a comparatively large condenser due to the diffusion of a modest amount of permeate in a significantly larger volume of sweep gas [86]. In addition to the above-mentioned configurations, a combination of AGMD and SGMD, called thermostatic sweeping gas membrane distillation, was also developed [101]. In this case, the inert gas is passed through the gap between the membrane and the condenser, where a part of the vapor is condensed. The remaining water vapor is condensed outside the membrane module by an external condenser. In VMD design (Figure 6D), a pump is employed to create vacuum on the permeate side of the membrane, which enhances mass transfer across the membrane. Accordingly, higher permeate flux can be achieved compared with the other MD configurations. As shown in Figure 6D, the hot feed solution is in direct contact with the upstream side of the hydrophobic membrane and vapor-liquid interface is formed at the entrance of the membrane pores. Hot water evaporates at the vapor-liquid interface, and the vapor is condensed outside the membrane module resulting in negligible heat loss of the system, which is another great advantage of this configuration. Likewise, Hendren et al. [95] indicated that the loss of performance associated with the heat transfer in DCMD using a ceramic membrane could be resolved in the VMD process. The other advantage of this technique is its good integration with other industrial processes, such as UF [102], NF [103] and RO [104] for superior water recovery.

To the best of our knowledge, there is no prior work related to the application of ceramic membrane distillation for the treatment of mine effluent and VMD. Accordingly, the performance of modified ceramic membranes for salt rejection from synthetic water will be discussed in this section.

Cerneaux et al. [100] compared the performance of two tubular ceramic membranes, one made of zirconia and another of titania, for MD desalination purpose under various configurations; namely, DCMD, AGMD and VMD. The ceramic membranes were chemically grafted by perfluoroalkylsilane molecules (C8) in order to induce hydrophobicity. The performance of the membranes was evaluated in desalination of 0.5 M (29.2 g/L) and 1 M (58.4 g/L) NaCl solutions. High rejection rates (>99%) were obtained applying a titania ceramic membrane with a pore diameter of 5 nm in VMD, AGMD as well as DCMD. The permeate flux was much lower in DCMD and AGMD (0.8 LMH) compared to VMD (6.1 LMH). In addition, feed temperature dramatically affected the permeate flux in DCMD and AGMD such that the permeate flux of 0.8 LMH was only achieved at a high feed temperature of 95 °C. Regarding the zirconia membrane, with a pore diameter of 50 nm, more than 99% rejection rate was obtained in DCMD and AGMD during the entire course of the experiment whereas the rejection rate gradually decreased with time in VMD (i.e., from 99% to 96% after 4.5 h of experiment). This was attributed to the deposition of salt on the membrane surface. The highest flux was also reached under this condition although it consistently diminished during the experiment (from 12.1 to 7.5 LMH), further indicating the fouling of the membrane. In addition, concentration polarization effect increased by raising the salt concentration in the feed solution over time. The lowest flux was measured in DCMD due to the higher temperature polarization. It is worth noting that fouling was not observed in DCMD and AGMD experiments. It was also revealed that the membrane pore size and porosity are the most significant parameters in determining the permeate flux, although both membranes had the same LEP. Table 2 presents a summary of the studies that have dealt with the application of ceramic membranes in VMD systems.

Fang et al. [105] developed a hydrophobic alumina hollowfiber membrane with an average pore size of 700 nm, suitable for MD application. The membrane was grafted with fluoroalkylsilane (FAS) and was tested in a VMD desalination system. In this regard, the shell side of the fibers was exposed to a 40 g/L NaCl solution at 80 °C and a vacuum pressure of 0.04 bar was applied on the lumen side of the fibers. A relatively high permeate flux of 42.9 LMH was obtained with over 99.5% salt rejection, comparable with the best performance of polymeric membranes. However, the permeate flux and salt rejection gradually decreased over the time, which might be due to the supersaturation of the solution, leading to the precipitation of salt on the membrane surface. The precipitated salt on the pore mouth introduces additional resistance to the permeation of the water vapor, thereby diminishing the permeate flux. It could also reduce the hydrophobicity of the membrane, resulting in a decline in the salt rejection. After a simple washing-drying step, the desalination performance was recovered effectively [105]. In very recent studies, Ko et al. [106,107] fabricated alumina hollow fiber membranes with smaller pore size (220 and 165 nm) compared to the previous study (700 nm) and tested their potential applicability for MD by using 0.6 [106] and 1.0 M [107] NaCl solutions. The membranes were modified with FAS to render them hydrophobic. Their results demonstrated that a high permeate flux up to 60 LMH and salt rejection over 99.9% could be achieved in a VMD process. This permeation rate is one order of magnitude higher than that of tubular ceramic membranes reported by Cerneaux et al. [100], likely owing to the unique microstructure of the ceramic hollow fiber with high porosity, low tortuosity and the low thickness of the membrane. In addition, rising the temperature of the feed solution from 50 to 60 and 70 °C successively increased the permeate flux due to the increment in mass transfer driving force (i.e., vapor pressure gradient). The flux remained constant during the 4.5 h operation with no evidence of membrane fouling.

Zhang et al. [108] reported similar results by adopting a silicon nitride hollow fiber membrane, which was grafted with FAS for VMD application. This non-oxide ceramic membrane exhibited high hardness and mechanical strength as well as excellent resistance to oxidation, thermal shock and corrosion. The applicability of the developed membrane for VMD was tested with 20 and 40 g/L NaCl solutions. Relatively high permeate fluxes were measured, which decreased by increasing the salt concentration in the feed solution, as expected (22.3 vs. 25 LMH). The rejection efficiency reached over 99% in both cases. In another study by the same researchers, the short-term performances of VMD and DCMD configurations were compared with respect to salt rejection and permeate flux [118]. Around three times higher permeate flux was achieved in VMD process than that of DCMD, mainly due to the temperature polarization effect in DCMD, although salt rejection was found to be over 99% in both cases. In addition, superior long-term stabilities, in terms of the salt rejection and water flux, were demonstrated in VMD. The salt rejection and permeate flux only slightly declined after 24 h of operation due to the crystallization of NaCl on the membrane surface, which could partially block the membrane pores and weaken the hydrophobicity of the membrane. A simple water washing and drying step recovered the membrane performance [118]. These results imply the promising candidature of silicon nitride ceramic membrane for the industrial application of VMD system. In addition to the study by Zhang et al. [118], other research works have since corroborated that ceramic membranes exhibit superior performance in VMD applications relative to DCMD, and that ceramic membranes are not yet competitive in DCMD with polymeric membranes [109,113].

Wang et al. [109] developed a microporous β-Sialon ceramic hollow fiber membrane, which consists of a mixture of silicon nitride and alumina powders. The developed membrane exhibited a much lower thermal conductivity compared with silicon nitride or alumina membranes, which makes it interesting for MD application. After surface modification by grafting FAS, satisfactory NaCl rejection and permeate flux were attained, as indicated in Table 2.

In the above-mentioned studies, the pore size control of the modified membrane was executed by sintering process, requiring high temperature or pressure, and therefore it is difficult to modify the membrane with good pore size control. In this regard, Huang et al. [110] developed a simple coating method to create a superhydrophobic solid layer on a ceramic alumina membrane. Accordingly, a mixture of silica/alumina nanoparticle and a FAS/ethanol solution was applied to coat alumina disk membranes with a pore size of 2.4 μm. The modified membrane had a high contact angle of 158° and an average pore size of 0.4 μm in the coating. The performance of the coated membrane adopted in a VMD system was examined using a 35 g/L NaCl solution. A high rejection rate of 99.9% and permeate flux of 29.3 LMH were achieved at a feed solution temperature of 70 °C. It was also found that increasing the feed flow rate from 30 to 60 L/h notably increased the permeate flux from 18 to 29.3 LMH, respectively. Further increment in the feed flow rate, however, did not have any impact on permeate flux, most likely due to the limitation of the membrane. The permeate flux was also influenced by salt concentration at concentrations above 15 g/L. Nevertheless, rejection performance of 99.9% was attained under all conditions and the flux remained constant over the two hours experimental course, except for a small reduction at the onset of the experiment due to the concentration/temperature polarization. The drawback of this modification is the possible release of nanoparticles from the coating after long-term operation of the membrane, resulting in membrane pore blockage or contamination of the treated water [110].

Although fluorinated silanes are the most common modifying agents for rendering ceramic membranes hydrophobic, a need has emerged for non-fluorinated alternatives, since fluorinated substances are known for negative environmental impacts and their regulation is becoming increasingly stringent [119]. Hydrophobic ceramic membranes with high distillation performance have also been obtained using fluoride-free agents [111]. Accordingly, Chen et al. [111] reported a highly hydrophobic tubular alumina membrane grafted by hexadecyltrimethoxysilane in ethanol solution. Visual difference was not found between the surface of the ceramic membrane before and after modification. Examinations of the water contact angle revealed the highly hydrophobic characteristic of the modified membrane (contact angle > 150°). This study demonstrated that although the thickness of the ceramic membrane’s active layer slightly affects the performance of the membrane in VMD, support layer of the membrane is the main contributor to the total mass transfer resistance. Consequently, reducing the mass transfer resistance in the support layer (e.g., by increasing the pore size and porosity or reducing the thickness) is crucial to enhance the permeate flux of ceramic membranes in the VMD process. The membranes with different active layer pore size (150 nm vs. 480 nm) exhibited a high retention of NaCl close to ~99.9% over 5 h experimental course (the concentration of NaCl in the feed solution was 30 g/L). The surface of the membrane with larger pore size was partially covered by precipitated salt after the VMD experiment due to supersaturation of the salt solution at pores larger than 500 nm, leading to a decrease in the hydrophobicity of the membrane, which could cause a decline in salt rejection after long-term operation. The long-term results of ceramic membranes with 150 nm pore size in VMD process indicated a stable rejection performance (99.9%) and permeate flux (30 LMH) during 18 h experimental period. Moreover, the microspectroscopy images of the membrane at different stages of the experiment revealed an excellent stability of the grafted alumina membrane. The results of this alumina tubular membrane are comparable with silica/alumina nanoparticle-FAS covered alumina disk membranes [110].

Alkylsilanes, like the grafting agent used by Chen et al. [111], are non-fluorinated silanes in which the organic functional group is a straight-chain hydrocarbon. Naturally, the length of this hydrocarbon chain influences the hydrophobicity of ceramic membranes modified with alkylsilanes. Pagliero et al. [112] investigated the use of methylchlorosilane (MTS) in VMD of NaCl solutions. MTS is an alkylsilane with the shortest hydrocarbon chain possible, since the functional group is a methyl (−CH_3_) group. Such a short hydrophobic chain was chosen to mitigate effects of grafting on the pore size and geometry of the membranes. Using alumina membranes with pore sizes of 70 and 200 nm, contact angles after modification increased up to 145°, indicating that even with very short alkyl chains, the membranes were rendered adequately hydrophobic for MD applications. In the treatment of solutions containing 90 g/L NaCl, distillate fluxes in the range of 20–30 LMH and rejections >99.9% were reported. In comparison to a commercial polymeric membrane that was used as a reference, these flux values were found to match, and even surpass in some cases, the performance of the polymeric membrane.

An important aspect of ceramic VMD that is often overlooked is the stability of the modified hydrophobic surfaces in hot and highly saline environments. Schnittger et al. [114] sought to investigate this by testing alumina (400 nm pore size) and titania (200 nm pore size) membranes in VMD of highly saline mixtures. The membranes were modified using FAS as well as non-fluorinated n-octyltriethoxysilane. The modified membranes exhibited no loss in LEP during long-term exposure to highly concentrated NaCl solutions of 250 g/L at 100 °C. During VMD tests with solutions of 350 g/L, it was found that titania membranes outperformed alumina counterparts. This was attributed to superior mass transfer through the larger pore sizes of titania support layers, and the lower thermal conductivity of titania compared to alumina. A maximum permeate flux of 35 LMH was achieved in conjunction with rejections over 99.9%. The results suggested that the modified ceramic membranes were robust enough for use in highly saline and abrasive conditions, and that they can be competitive with established commercial polymeric membranes in VMD applications.

In ceramic MD applications, conventional and commercially available ceramic membranes are often used. These are typically made of common ceramic membrane materials such as alumina and titania. One of the drawbacks of ceramic membrane use is the increased manufacturing cost associated with these materials. As such, researchers have begun exploring the use of inexpensive inorganic materials to fabricate ceramic membranes. Zhang et al. [115] used coal fly ash (CFA) to develop a ceramic membrane for VMD. The membranes were rendered hydrophobic using a fluorinated chlorosilane. Four types of CFA membranes with different pore size distributions were synthesized, with mean pore diameters ranging from 0.15 to 2.05 µm. In treating 10,000 ppm NaCl solutions, results of the study showed that when the mean pore size increased from 0.15 µm to 1.57 µm, flux increased from 5.15 LMH to 19.72 LMH, but at the expense of salt rejection (99.95% vs., 99.87%). Furthermore, in a comparison test between a CFA membrane (0.18 µm pore size) and an alumina membrane (1.57 µm pore size), the CFA membrane offered a higher water flux (9.54 LMH vs. 6.62 LMH) despite its reduced pore size. In this test, salt rejection for the CMA membrane was 98.36%. These results demonstrate that alternative inorganic materials like clays and silicates are promising candidates for inexpensive ceramic membrane fabrication, but synthesis methods still need to be refined to yield membranes that can consistently offer high fluxes and salt rejections (>99.9%) akin to those seen with modified commercial ceramic membranes.

The vast majority of literature on ceramic membrane MD applications, including the studies discussed thus far, focuses on the desalination of NaCl solutions. However, a select few research works have instead investigated ceramic VMD for the concentration of other types of aqueous streams. Chen et al. [116] developed a hybrid nanofiltration-VMD process for the treatment of simulated radioactive wastewater to recycle boric acid [116]. Nanofiltration was first used to reject impurity ions Co^2+^ and Ag^+^ with efficiencies of >99.9% and >95%, respectively. For VMD, ceramic membranes were rendered hydrophobic by grafting with hexadecyltrimethoxysilane, as per the author’s previous work [111]. VMD in this application maintained a flux above 20 LMH while rejecting >99.9% of boric acid, concentrating it from 1 to 107 g/L.

VMD with ceramic membranes has also been applied for the removal of volatile organic compounds (VOCs) from water. Kujawski et al. [93] modified titania, alumina and zirconia membranes with FAS for use in the separation of ethanol (EtOH), ethyl acetate (EtAc) and butanol (BuOH) from water. Membranes with two different pore sizes were tested (5 kDa and 300 kDa). It was found that membrane selectivity in the separation of water-VOC mixtures strongly depended on modified membrane pore size. The smaller pore size membrane was less efficient in separating VOCs from water with an EtAc separation factor of 1.3–30 compared to the larger pore size membrane (EtAc separation factor of 32–60). Both membranes, however, were less effective than hydrophobic polymeric membranes (EtAc separation factor of 25–450). The authors later built upon this work by using non-fluorinated alkylsilane modifying agents and using methyl-tert-butyl ether (MTBE) in addition to BuOH and EtAc as VOCs in water [117]. The hydrophobized membranes were once again shown to have high efficiency in removing VOCs from binary aqueous solutions via VMD. The highest performance was obtained in the separation of MTBE and EtAc as VOCs. Through various kinetic studies, it was elucidated that at the smaller pore size of 5 kDa, selectivity depended greatly on the type of membrane modifying agent used and the separation mechanism thus followed the solution-diffusion model. Contrarily, for the larger 300 kDa membranes, no impact of surface chemistry on separation properties was observed, indicating that these membranes simply act as a physical barrier and the separation process is controlled by liquid-vapor equilibrium. In additional studies [120], the authors demonstrated that non-fluorinated alkylsilanes can be in fact more suitable for VOC separation by ceramic VMD than their fluorinated counterparts with regard to desirable surface properties and separation performance.

An important caveat to note is that, because membrane distillation fluxes are driven by vapor pressure differences between the feed and permeate membrane surface, the driving force for the process could potentially decrease significantly along the membrane length if there is a large decreasing temperature gradient between the inlet and outlet of the membrane module. It is thus imperative to consider membrane length when comparing the fluxes reported by different studies. In the surveyed studies, the highest flux of 60 LMH was reported by Ko et al. [106] with FAS-alumina hollow fiber membrane and attributed to the ultrathin wall thickness (0.2 mm) In their study, the authors utilized membranes with lengths of 9 cm, which is at the lower extremity of the range of membrane lengths reported in the reviewed studies (8–50 cm) as seen in Table 2. Shorter membrane lengths will naturally lead to lower temperature decline along the membrane module and maintain a more consistent driving force, thus leading to higher fluxes. Commercially available pilot-scale ceramic membranes can possess lengths upwards of 1 m, which would result in lower fluxes compared to the smaller membranes tested in the surveyed literature if operated under similar conditions. One possible method to alleviate the temperature gradient along the membrane length is to operate at higher feed flow rates, which helps maintain more consistent heat transfer in the membrane module. Therefore, both the membrane length and feed flow rate or crossflow velocity are included in Table 2 to better compare fluxes between different studies. In tubular ceramic membrane modules, the feed solution commonly flows inside the tubes and a vacuum is applied on the shell side of the system. The typical advantages of tubular membrane modules over hollow fiber modules are consistent heat and mass transfer (which could help minimize driving force decline along membrane length), uniform plug flow of liquid in the channels, lower membrane fouling and easier cleaning and reuse [121].

Membrane fouling is one of the main obstacles hindering large-scale industrial application of MD. Like all other membrane processes, fouling causes a decline in the membrane permeability owing to the deposition of contaminates on the membrane surface and/or inside the membrane pores, which poses additional resistance to mass and heat transfer. Moreover, although, theoretically, only water vapor is allowed to pass through the membrane pores, several factors, such as poor long-term hydrophobicity of the membrane, very thin thickness of the membrane and membrane damage and degradation, could lead to deposition of foulant and consequently wetting of the pores, which can reduce salt rejection and deteriorate the MD performance [122]. Understanding the fouling mechanism is essential to come up with an appropriate approach towards preventing, minimizing and mitigating the fouling formation as well as membrane cleaning. Generally, the following factors govern the formation and the extent of fouling: (1) foulant characteristics (concentration, solubility, molecular size, diffusivity, charge and hydrophobicity); (2) membrane properties (hydrophobicity, pore size and pore size distribution, surface charge, surface roughness and surface functional group); (3) operational conditions (flow velocity, solution temperature, flux and vacuum pressure for the case of VMD); (4) feed water characteristics (pH, solution chemistry, ionic strength and presence of organic/inorganic matters). In addition, the MD configuration may also affect membrane fouling. Chiam and Sarbatly [121] found that among various MD systems, VMD is more subjected to fouling problems because of the greater pressure gradient across the membrane. The membrane fouling in MD systems can be divided into organic fouling (e.g., humic acid, fulvic acid, protein, polysaccharides and polyacrylic polymers), inorganic fouling (e.g., calcium carbonate, calcium sulfate, silicate, NaCl, calcium phosphate, barium sulfate, strontium sulfate, ferric oxide and aluminum oxide) and biological fouling (e.g., bacteria and fungi, sludge, algae, yeast), which typically occurs simultaneously.

Water evaporation and temperature changes in MD process causes supersaturation condition, which lead to nucleation and growth of crystals on the membrane surface. This type of inorganic fouling should be rigorously considered in determining the severity of fouling in a system concerning mine discharge treatment. It was found that calcium crystals are responsible to initiate the precipitation of the other salts on the membrane surface [104]. Scales were not only form on the membrane surface, but may also form inside the membrane pores, leading to damage of the membrane [123]. These deposits usually start forming at the biggest pores of the membrane where are prone to accelerated wettability [122]. Several factors influence the scaling rate including flow condition, solution temperature, membrane surface roughness and morphology, surface material, degree of supersaturation, and the presence of particulates and impurities in water [122]. It was found that reducing the feed water temperature and pH, and increasing feed velocity could limit the precipitation of calcium carbonate, which is one of the main scaling initiators [124]. Another common scale is calcium sulfate or gypsum, which is the main concern during distillation of mining wastes due to the high SO_4_^2−^ concentration. Deposition of iron oxides, which are commonly present in particulate form, is also another concern towards the application of MD technology for treatment of mining effluent. In addition to inorganic fouling, adsorption/deposition of colloidal organic matters, such as humic substances and polysaccharides, which are present in source water (e.g., ground water), is another concern of MD systems. The extent of fouling by humic substances is affected by pH, ionic strength, concentration of monovalent and divalent ions, operating conditions, and membrane surface properties [122]. Hydrophobic characteristic of MD membranes favors adsorption of humic macromolecules on the membrane, especially under low solution pH and high divalent ions concentration. Biofouling formation in MD system is limited owing to the high salinity of the feed water as well as its high temperature, which is higher than the growth temperature of most bacteria [122]. Feed pre-treatment and regular membrane cleaning have been found to be effective techniques to mitigate and control membrane fouling [122]. Increase in feed velocity could enhance the shear stress on the surface of the membrane and alleviate the fouling of the membrane [125]. In addition, gas bubbling in the feed side maximizes shear stress at the membrane surface, and thus, minimizes the formation of deposits. It was also found that addition of antiscalants slows down the precipitation of crystals. However, it may also reduce the hydrophobicity of the MD membrane and exacerbate fouling if the dosage is not optimized [126]. Therefore, care must be taken with the use of antiscalants.

Fouling studies discussed here have been conducted using polymeric membrane, and the long-term performance of ceramic membranes in MD systems as well as their fouling propensity have remained unknown. In addition, despite the potential application of ceramic membrane in VMD systems for treatment of mining discharge and AMD, it has not been investigated to the best of our knowledge. Accordingly, future research should focus on analyzing the role of different parameters, optimizing the process performance and determining the long-term outcomes of ceramic VMD process for treatment of mine waste with respect to water recovery, membrane fouling and rejection of different salts, heavy metals and other dissolved, colloidal and particulate contaminants. In addition, examining the potential application of RO-VMD hybrid process for high recovery of water from mine waste is of great interest.

## 7. Concluding Remarks and Future Directions

The limitations facing the conventional treatment methods as well as the stringent regulations for the quality of mining discharge and AMD highlight the need for effective treatment technologies with minimum environmental impacts. In this regard, the application of ceramic membrane technology is valuable owing to its robustness with respect to the treated water quality, small process footprint, superior chemical, thermal and mechanical stability, long lifespan under aggressive operating conditions and ease of cleaning. Despite this fact, the review of the previous studies revealed that the number of investigations dealt with the application of ceramic membranes in the mining sectors is limited. This might be related to the complex nature of mine discharge water and AMD. Due to the ongoing importance of ceramic membrane processes for treatment of mining discharge, future research efforts should further focus on the potential application of various ceramic membranes with different pore size either stand alone or integrated with other technologies in treatment of mining effluents, with a particular emphasis on recycling process water in mineral processing operations. Based on this review, suggested future research are summarized below:

- In-dept characterization of mining discharge and AMD prior to the development of an appropriate treatment train integrating ceramic membrane technology. Due to the significant impact of natural organic matter on the solubility of the dissolved metal species, performance of pretreatment processes, destabilization of colloids, membrane surface charge as well as membrane fouling and its reversibility, their concentration and nature need to be defined prior to the selection of the appropriate process train. Improved water characterization will aid in the selection of important process parameters such as required pre-treatment options to facilitate downstream membrane filtration, appropriate membrane pore sizes for rejection of targeted species, and membrane materials that could help mitigate interaction with potential foulants.
- Design and implement pilot-scale mining effluent and AMD treatment processes using various types of ceramic membranes with different pore sizes, either stand-alone or integrated with other treatment techniques. Pilot-scale testing is necessary to demonstrate the scalability of ceramic membrane processes in treating mine effluents. Process intensification and hybrid process development should be explored as a means of improving the economics of water treatment in this application
- Conduct long-term operation of pilot-scale ceramic membrane process to guarantee steady-state performance of the system without any or minimum requirement for membrane replacement. It is important to assess the longevity of ceramic processes and demonstrate that they are capable of yielding high flux and separation performance over extended periods of time. Their long-term performance will help inform the type and frequency of fouling remediation techniques (e.g., Backwashing, CIP) that are to be applied. The effect of repeated membrane cleaning on membrane durability over time should be closely established.
- Address pretreatment technologies that can be integrated with MF/UF/NF ceramic membranes for treatment of mining effluents and water recycling during mineral Processing (e.g., Floatation). High-level understanding of retention and fouling mechanisms associated with each treatment train as well as characterization of both membrane and foulants (such as surface charge, fractal dimensions and particle size) are crucial for selecting an appropriate treatment strategy for each specific water composition.
- Investigate the rejection performance of various ions, especially toxic heavy Metals (e.g., Arsenic and mercury) as well as sulfate, using modified/unmodified ceramic NF membranes for mine effluent and AMD treatment under different operations (permeate recovery, crossflow rate, operating pressure and temperature) and water quality Conditions (i.e., Ph, ionic strength, and concentration of inorganic and organic salts).
- Investigate the role of different Parameters (e.g., Feed water characteristics, membrane properties, modifying agent and operating condition), optimize the process performance and determine the long-term outcome of ceramic VMD process for treatment of mine waste and water recycling during mineral processing with respect to water recovery, membrane fouling and rejection of different salts, heavy metals and other dissolved, colloidal and particulate contaminants. There is currently a lack of available literature on the performance of VMD in the treatment of mine effluents, even though the technology has great potential applications in the recovery and reuse of resources from AMD, mineral Processing (e.g., Water recycling in floatation), sludge dewatering and maximizing the overall water recovery in mining industries
- Examine the potential application of RO-VMD hybrid process for high recovery of water from mining wastes. VMD is currently hindered by the technology’s need for a consistent heat source to operate effectively. Coupling it with mature industrialized technologies like RO could greatly improve both technical feasibility and process economics.
- Optimize the process design to reduce capital and operating costs and maximize water recovery. In addition, a comprehensive Cost–Benefit analyses comparing ceramic membranes with conventional treatment technologies should be the focus of future study. This entails assessing the long-term operational efficiency, membrane lifespan, and maintenance requirements under real-world mining conditions. Such analyses are essential for supporting informed decision-making and accelerating the industrial-scale adoption of ceramic membranes.
- Conduct systematic comparison of MF/UF/NF/VMD performance using polymeric and ceramic membranes, with respect to contaminant removal, membrane fouling, reversibility of fouling and techno-economic analysis. It is important to not only demonstrate the technical and economic feasibility of ceramic membranes in the treatment of mine-impacted water, but to also show that their performance is competitive, if not superior, to that of conventional polymeric membranes.
- Investigate the environmental impact and end-of-life management of ceramic membranes to better assess their long-term sustainability in mining applications. Studies that lead to information pertaining to the end-of-life management of ceramic membranes, such as recycling or disposal, should be conducted. This would help inform how to minimize the potential negative environmental concerns.

## Figures and Tables

**Table 2:** A summary of the studies on the application of ceramic membrane in VMD systems.

Feed Source and Characteristics	ΔT (°C)	Membrane Material	MWCO/Pore Aize	Vacuum Pressure (Bar)	Grafted Chemical	Feed Velocity	Permeate Flux (at Steady State)	Rejection Efficiency	Ref.
NaCl solution: 0.5 M (29.2 g/L)	40-ambient	Tubular zirconia Length: 15 cm ID: 7 mm OD: 10 mm	50 nm	0.003	Perfluoro-alkylsilane	210 L/h	Consistent decrease from 12.1 to 7.5 LMH	99–96%	[100]
NaCl solution: 0.5 M (29.2 g/L)		Tubular titania Length: 15 cm ID: 7 mm OD: 10 mm	5 nm				6.1 LMH	>99%	
NaCl solution: 1 M (58.4 g/L)							4.2 LMH		
NaCl solution: 0.68 M (40 g/L)	80-ambient	Hollow fiber alumina Length: 10 cm	700 nm	0.04	FAS	N/A	42.9 LMH	>99.5%	[105]
NaCl solution: 0.60 M (35 g/L)	70-ambient	Hollow fiber alumina Length: 9 cm	220 nm	0.03	FAS	60 L/h	60 LMH	>99.9%	[106]
			165 nm				30 LMH		
NaCl solution: 1 M (58.4 g/L)	50-ambient	Hollow fiber alumina Length: 25 cm	220 nm	0.03	FAS	8.4 L/h	20 LMH	>99.9%	[107]
NaCl solution: 0.34 M (20 g/L)	70-ambient	Hollow fiber silicon nitride Length: not given	740 nm	0.02	FAS	N/A	25 LMH	>99%	[108]
NaCl solution: 0.68 M (40 g/L)							22.3 LMH		
NaCl solution: 0.34 M (20 g/L)	50-ambient	Hollow fiberβ-Sialon (ɑ-Si_3_N_4_ + Al_2_O_3_) Length: 8 cm	800 nm	0.02	FAS	100 L/h	4.0 LMH	>99%	[109]
	80-ambient						12.2 LMH		
NaCl solution: 0.68 M (40 g/L)	50-ambient						3.7 LMH		
	80-ambient						10.7 LMH		
NaCl solution: 0.60 M (35 g/L)	70-ambient	Alumina disk Length: n.a.	2.4 um (400 nm after modification)	0.03	Silica/alumina nanoparticle + FAS/ethanol	60 L/h	29.3 LMH	99.9%	[110]
						30 L/h	18 LMH		
NaCl solution: 0.51 M (30 g/L)	70-ambient	Tubular asymmetric alumina Length: 11 cm	Active layer: 150 nm Support layer: 3.2 um	0.05	Hexadecyltrimethoxysilane	160 L/h	30 LMH	99.9%	[111]
NaCl solution (90 g/L)	70-ambient	Tubular alumina Length: 9.2–11.9 cm	70 nm	0.02	Methyltrichlorosilane (MTS)	1.4 m/s	21.5–23.1 LMH	99.9%	[112]
			200 nm				29.1–31.2 LMH		
NaCl solution (30 g/L)	60-ambient	Tubular alumina	100 nm	0.1	FAS	0.7 m/s	16 LMH	99.9%	[113]
			400 nm				13 LMH		
		Tubular titania	100 nm				17 LMH		
			400 nm				25 LMH		
		Tubular zirconia Length: 25 cm	110 nm				6 LMH		
NaCl solution (350 g/L)	55 to 75-ambient	Tubular alumina	100, 200 and 400 nm	0.075 to 0.125	FAS and n-Octyltriethoxysilane	0.14 to 1.08 m/s	17.5 LMH	>99.9%	[114]
		Tubular zirconia Length: 25 cm					35 LMH		
NaCl solution 10,000 ppm (~10 g/L)	55 to 70-ambient	Tubular coal fly ash (CFA) Length: 50 cm	0.15 and 0.18 µm	N/A	FAS	40 to 80 L/h	5–20 LMH	98–99.9%	[115]
Simulated radioactive wastewater (0–40 g/L boric acid, 0–200 ppm Co^+^ and Ag^+^)	60 to 80-ambient	Tubular alumina Length: 11 cm	200 nm	0.85 to 0.95	Hexadecyltrimethoxysilane	40 to 160 L/h	15–35 LMH	99.9% (boric acid rejection)	[116]
Water-organic solvent mixture 0–20 wt% EtOH 0–4 wt% EtAc 0–4 wt% BuOH	35-ambient	Tubular titania, zirconia and alumina Length: 15 cm	5 kDa	0.04	FAS	N/A	N/A	EtAc separation factor 1.3–30	[93]
			300 kDa					EtAc separation factor 32–60	
Water-organic solvent mixture 0–3 wt% BuOH, EtAc and MTBE	25 to 65-ambient	Tubular titania Length: 15 cm	5 kDa	0.001	FAS and n-Octyltriethoxysilane	17 L/min	N/A	Separation factor: 5–56 (MTBE 1–30 (EtAc) 1–7 (BuOH)	[117]
			300 kDa					Separation factor: 62–109 (MTBE) 35–48 (EtAc) 9–12 (BuOH)	
			300 kDa						

## Data Availability

No new data were created or analyzed in this study. Data sharing is not applicable to this article.

## Abbreviations

The following abbreviations are used in this manuscript:

AMD	Acid Mine Drainage
MF	Microfiltration
UF	Ultrafiltration
NF	Nanofiltration
RO	Reverse Osmosis
VMD	Vacuum Membrane Distillation
HDS	High-Density Sludge
MWCO	Molecular Weight Cut-Off
IEP	Isoelectric Point
TMP	Transmembrane Pressure
TSS	Total Suspended Solids
TOC	Total Organic Carbon
MD	Membrane Distillation
LEP	Liquid Entry Pressure
PTFE	Polytetrafluoroethylene
PP	Polypropylene
PVDF	Polyvinylidene Fluoride
DCMD	Direct Contact Membrane Distillation
AGMD	Air Gap Membrane Distillation
SGMD	Sweeping Gas Membrane Distillation
FAS	Fluoroalkylsilane
CFA	Coal Fly Ash
VOCs	Volatile Organic Compounds
MTBE	Methyl-tert-butyl Ether
